# “Privileged” under Nazi-Rule: The Fate of Three Intermarried Families in Vienna

**DOI:** 10.1080/14623528.2019.1634908

**Published:** 2019-07-03

**Authors:** Michaela Raggam-Blesch

**Affiliations:** Institut für Zeitgeschichte, University of Vienna, Wien, Austria

**Keywords:** Vienna, Austria, Holocaust studies, microhistory, oral history, intermarried families, mixed marriages, Jewish-Christian relations, postwar Austria

## Abstract

In this article, I highlight the daily life of three intermarried families in Vienna during the interwar years, the Nazi oppression and the immediate postwar period. All three families led secular lives with varying ties to their Jewish and non*-*Jewish environment. After the Nazi takeover in March 1938, intermarried families along with the Jewish population experienced immediate discrimination and ostracism. This paper aims to outline how the Nazi takeover affected these families in their day-to-day encounters with non*-*Jews as well as their relationships with friends and family members. “Mixed marriages” and their families navigated between Jewish and non-Jewish worlds, usually not fully belonging to any side. Thus, most of them experienced social isolation and a lacking sense of belonging, while others – mostly younger generations – sometimes found new forms of community. During the last years of the war their protection became more precarious and even trivial infractions against Nazi laws could lead to imprisonment and deportation. Since “mixed marriages” and their families did not officially learn about the key factors of their safeguarding, they were left to their own instincts on how to uphold their protection.

## Introduction

A few years after the end of the Second World War, *Mathilde Hahn*,^1^ a woman of Catholic descent, took her own life. After having stood by her Jewish husband and child through years of ostracization and hardship, enabling their survival during the Nazi regime, she simply could not go on any longer: According to her daughter, the degradation had worn her out.^2^ Sometime earlier, already in November of 1945, Oskar Baader committed suicide. He too had upheld the sole responsibility of protecting his Jewish wife during the Nazi years in Vienna, while being deprived of his position as a high school teacher due to his Jewish spouse. In the fall of 1944 he had been drafted to a forced labour deployment in the Organization Todt (OT), where Baader – a veteran officer of the First World War – endured the demeaning treatment of his OT-commanders. According to his son Gerhard, this turned out to be the ultimate humiliation he would never recover from: “He only came home to die.” He found his final resting place in the Baumgarten cemetery on the outskirts of Vienna.^3^

After the war, intermarried families^4^ were considered the privileged ones, those who had been lucky enough to remain in Vienna while everyone else was being deported, most of them to their imminent deaths. Compared to the horrors of the camps, they were the ones to whom “nothing had happened.” In this article, I highlight the daily life of “mixed marriages” and their families during the years of the Nazi regime in Vienna and the hardships they endured. While each of them formed the nucleus of an “emotional community” (Rosenwein),^5^ they did not necessarily form bonds of solidarity with each other during times of deep emotional upheaval.^6^ By focusing on the biographies of three families, I will provide insights into individual responses to persecution and their interactions with their Jewish and non*-*Jewish environment over a longer period of time, including the pre- and postwar period. Since this article is based on ordinary people, there is little official documentation available to rely on, apart from family documents. Therefore, the main primary sources for this paper are oral history interviews conducted with the children of three families in the late 1990s and early 2000s^7^ as well as a short autobiographic manuscript.^8^ While oral history interviews are historical sources produced by the research interest of organizations or individuals decades after the events took place, often containing inaccuracies regarding historical data, their subjective nature can be made productive with a clear awareness about which research questions can be answered. Whereas exact dates, the chronology of persecution measures and other determinants of the historical context can be researched through other sources, oral history interviews and testimonies are particularly valuable sources for exploring coping strategies as well as aspects of identity and belonging.^9^ The high demands on survivors such as stating the correct colour of uniforms or naming exact dates – sometimes even in settings where they had been lacking the means to trace time – has been shown with court testimonies in postwar trials against perpetrators.^10^ At the same time, even “objective” sources such as contemporary historic documents were created in a specific context with an intrinsic agenda that requires contextualization as much as the specific setting of oral history interviews. The reconstruction of events of the past from the perspective of the present sometimes allows sensitive topics that had been difficult to communicate in earlier years to emerge with grater candour in later testimonies, as Christopher Browning has shown.^11^ Following Saul Friedländer’s concept of writing the history of the Shoah as an *integrated* history, the persecuted are taken seriously as individuals and their perspective is taken into account as much as the actions of the perpetrators. Autobiographical sources and oral history interviews reveal insight into individual survival strategies and represent an important corrective to the sources produced by the Nazi state authorities. They offer the chance to discover small acts of resistance or self-assertion that were not reflected in the sources created by the perpetrators. Victims thereby cease to appear as an anonymous collective and their agency and strategies of survival become visible.^12^ Furthermore, the microhistorical lens, as Claire Zalc and Tal Bruttmann have pointed out, encourages us to take a closer look at the particularities of individual biographies in order to gain a deeper understanding not only of regional differences, but also of the multi-layered realities of the Holocaust.^13^

While this paper focuses on Vienna,^14^ the situation of intermarried families is comparable with Germany, since most edicts constricting the daily life of this group pertained to all “mixed marriages”^15^ and their families in the *Altreich* (Germany) and the *Ostmark* (Austria).^16^ Nevertheless, regional differences between cities – depending on the local Nazi officials in charge – could at times be significant, as Monica Kingreen has shown with the deportation of Jewish members of “mixed marriages” in Frankfurt am Main.^17^

Conversely, early Holocaust research considered the persecution of *Mischlinge* ^18^ and “mixed marriages” as marginal.^19^ Only in the late 1980s – starting with the first publications of Ursula Büttner and Jeremy Noakes, followed by the comprehensive study by Beate Meyer and the publication of James F. Tent – the fate of intermarried families during the Nazi regime gained increasing attention.^20^ In addition, the publication of Victor Klemperers diaries, who survived in a “mixed marriage” in Dresden, further sparked public and scholarly interest in the subject matter.^21^

In this article, three intermarried families in Vienna will be examined during the time span from the interwar years through the Nazi oppression to the immediate postwar period. All three families led secular lives with varying ties to their Jewish and non*-*Jewish environment.^22^ After the so-called *Anschluss* ^23^ to Nazi Germany in March 1938, intermarried families along with the Jewish population experienced immediate discrimination and ostracism. This article aims to outline how the Nazi takeover affected these families in their day-to-day encounters with non*-*Jews as well as their relationships with friends and family members. “Mixed marriages” and their families navigated between Jewish and non-Jewish worlds, usually not fully belonging to any side. Thus, most of them experienced social isolation and a lacking sense of belonging, while others – mostly younger generations – sometimes found new forms of community.

## Political Turmoil and Pervasive Antisemitism: The Interwar Years in Vienna, 1918*–*1938

The three Jewish protagonists that are at the focus of this paper stemmed from families that had moved to Vienna at the beginning of the twentieth century. While some of them came from more observant backgrounds, they all had somewhat distanced themselves from religion before they chose to marry a non*-*Jewish partner.

Cecilia Adler, born in 1887, was the eldest daughter of ten siblings of a Moravian Jewish family that moved to Vienna around 1900. After the early death of her father, a tailor, Cecilia helped her mother raise her younger siblings. Later on, she caught up with her education, studied for her final exams in evening classes and became a university student in the 1920s. During these years, the Vienna University was a hotbed for antisemitism.^24^ At the university, Cecilia met the Catholic Oskar Baader, a promising student of English literature, who was about to start an academic career.^25^ After their engagement, however, Oskar Baader was excluded from the influential German Academic Anglicist Association (DAA) due to his Jewish fiancé, since the DAA had an “Aryan paragraph” in their membership statutes.^26^ This put an end to his professional aspirations. He became a teacher at a Gymnasium (high school) instead. Their son Gerhard, born in 1928, was baptized after birth, yet grew up in a secular family with a strong socialist identity. While they always had a Christmas tree, Gerhard and his mother Cecilia also regularly attended family celebrations at the Adler family home during the Jewish holidays. The Adler family upheld Jewish traditions, which was not an expression of their religious adherence, since most of Cecilia’s siblings were also supporters of the Social Democratic party and her sister Henriette had married a non*-*Jew as well. The Baaders, as Social Democrats, were able to get an apartment in one of the new community buildings of the ground-breaking Red Vienna public housing projects. However, since their building was located in the bourgeois district of Hietzing, they hardly had any close contact with people in the neighbourhood – apart from an occasional playmate of Gerhard, who lived in the same building. They mostly socialized with other Social Democrats, who usually did not live in the area. While some of them might have been of Jewish descent, Gerhard Baader as a child perceived their circle of friends mainly as non*-*Jewish. The political changes in the wake of Austrofascism and the prohibition of the Social Democratic party in 1934 already affected the Baader family in the years leading up to the Nazi takeover in 1938. Gerhard Baader considers this time as the end of his childhood, since the family, whose allegiance lay with the forbidden Social Democrats, already had to be careful in their social encounters during the Dollfuss/Schuschnigg era.^27^

*Albert Hahn*,^28^ born 1892, stemmed from a Hungarian Jewish family. In the 1920s he was also enrolled at the Vienna University as a law student. Due to its high numbers of Jewish students, the faculties of law and medicine were particularly targeted during antisemitic riots.^29^ During this time, *Albert* became a member of the Zionist student fraternity *Kadimah*, which was founded to fight back antisemitism and gain pride in Jewish identity.^30^ This experience proved to be formative for *Hahn*. Even after his marriage to the Catholic *Mathilde Ruttner*,^31^ he maintained a strong Jewish and Zionist identity, which was somewhat exceptional among intermarried families. Their daughter *Judith* ^32^ was born in 1929. *Albert Hahn* made a point of sending her to a private girls’ school, the famous *Schwarzwaldschule*,^33^ where most of *Judith’s* classmates were of Jewish descent, in order to spare her the experience of antisemitism in the public school system. The family only had Jewish friends and *Judith* also joined the Zionist sports club *Hakoah* as a member of the swimming team.^34^

Moritz Freiberger, born in 1881, was one of the eldest siblings of seven, when his father passed away in the late 1890s. In order to help support his family, he started working in one of Vienna’s larger textile companies Blau. After getting his textile license he started his own wholesale business in yarn and fabrics and got married to the Catholic Mimi Löw. His widowed mother, however, never accepted her Catholic daughter-in-law, even though Mimi converted to Judaism prior to the wedding. As a result, Moritz Freiberger distanced himself from his Jewish roots and the family was rarely invited to Jewish family celebrations. This was not an unusual occurrence. In fact, most intermarried families faced reservations or even ostracism from non*-*Jewish as well as from Jewish family members.^35^ Moritz and Mimi Freiberger’s daughter Lotte, born 1923, therefore grew up in an acculturated Jewish family, who socialized mostly with non-Jewish circles. While receiving the obligatory religious education at school, she never attended a synagogue service together with her parents. Instead, the family celebrated Christmas, while the Jewish holidays were not kept.^36^ Compared to the Baader and *Hahn* family, the Freibergers belonged to the majority of Austrian intermarried families without strong religious or political convictions and with already loosened ties to the Jewish community.

## Life Changed Overnight: Intermarried Families after the *Anschluss*

On the eve of 10 March 1938, Moritz and Mimi Freiberger were glued to the radio, together with their fifteen-year-old daughter Lotte, listening with avid attention to Chancellor Kurt Schuschnigg’s radio address, when he uttered the famous farewell “God bless Austria” – yielding to the superior force of the Nazi regime. At this moment, it was clear, even to the teenage girl Lotte that something bad was about to happen, even though at this point nobody could really have fully anticipated the extent of what was about to take shape.^37^

The *Anschluss* to Nazi Germany radically changed the lives of the Jewish population and intermarried families. While anti*–*Jewish measures had progressed in Germany over the course of five years, they were implemented in Austria overnight. Images of the euphoric welcoming of Nazi troops by wide segments of the annexed Austrian population were contrasted by the sudden emergence of violence against Jews, most distinctly documented in photos of so-called *Reibpartien* (“cleaning squads”) – Jews kneeling on the ground and forced to scrub pro-Austrian slogans written in oil paint from the streets of Vienna, while being surrounded by gleeful onlookers.^38^ The violence of the *Anschluss* pogrom was a uniquely Austrian phenomenon: Not until the November Pogrom did the persecution in the *Altreich* (Germany) reach the levels of brutality seen in Austria.^39^ While the Jewish members of the Baader, *Hahn* and Freiberger family were lucky not to be caught in one of these events, they were nevertheless aware of the omnipresence of this humiliating practice and tried to avoid certain areas of the city.^40^

The *Nuremberg Laws* were retroactively out into effect as of 15 September 1935. Therefore, conversion to another religion after the *Anschluss* had no effect on a person’s “racial status.”^41^ The laws immediately affected intermarried families, who were a particular offense to the Nazi regime, since they betrayed the concept of a clear separation of a German *Volksgemeinschaft* of “Aryans” and the *Reich’s* Jewish population. While the *Nuremberg Laws* were a first attempt to define this group, questions about how to categorize and treat “mixed marriages” and their families continued to challenge National Socialist ideology and created discord among bureaucrats and policymakers.^42^ Efforts to introduce legal measures to dissolve “mixed marriages” were, however, met with caution by party leaders. Nevertheless, local Nazi authorities repeatedly put pressure on “Aryans” to leave their Jewish spouses and get a divorce.^43^

Within a short time after the *Anschluss*, intermarried families lost their livelihoods. Oskar Baader was discharged from his position as a high school teacher because of his marriage to a Jewish woman. At the same time, he was compelled by party members to get a divorce. His refusal to do so marked the beginning of the social isolation the family found itself in during the years to come. They had to live off Oskar Baader’s meagre pension and the few private lessons he was able to give.^44^ For the *Hahn* and Freiberger family, who were business owners, expropriation was imminent. *Albert Hahn’s* stationary store was seized shortly after the Nazi takeover. Moritz Freiberger, however, got support from an unexpected source: Two of his business colleagues – illegal members of the Nazi party prior to the *Anschluss* – used their newly gained power to intervene on his behalf in Berlin. They succeeded in getting him permission to continue his business as a former officer of World War One – something that rarely had an impact in Austria during this time. Nevertheless, in the wake of the November pogrom, permissions of this kind lost their bearing. On 10 November 1938, Freiberger’s wholesale yarn and fabric store, located in Hugo-Wolf-Gasse, just around the corner from the burning Schmalzhofgasse temple, was liquidated by party members of the nearby *Braunes Haus* in Hirschengasse.^45^ He was never to see any restitution for his lost business, since the store was not officially “Aryanized” and taken over but instead ransacked and closed altogether.^46^ Since the *Hahn* and Freiberger families lost their main source of income and only had limited access to their funds, their “Aryan” wives helped support the family with outwork jobs.^47^

## “Privileged Mixed Marriages” and “Non-privileged Mixed Marriages”

In order not to evoke objections from broad non-Jewish circles, Nazi authorities decided to provide privileged treatment to certain “mixed marriages.”^48^ Families like the Baaders, where the non-Jewish spouse was male (head of the household) or who had “half-Jewish” children who were baptized *(Mischlinge)*,^49^ were declared “privileged.” They received the same food ration cards as the general “Aryan” population. While “privileged mixed marriages” were usually able to remain in their apartments, Oskar Baader was nevertheless already expelled from his community building apartment together with his Jewish wife and son in December of 1938 – prior to the official revocation of Jewish tenancy rights in April 1939^50^ and in spite of being an “Aryan” himself. In fact, the Austrian Nazis in the Vienna municipality were eager to get a hold of apartments in the popular community buildings and expelled all Jewish tenants by the end of 1938.^51^ In addition, the local Nazi functionaries in Hietzing were particularly zealous to clear the bourgeois district from Jews. The Baader family thereafter found an apartment in the second district (Leopoldstadt) in a building that later became a *Judenhaus.* ^52^ Unlike other “privileged mixed marriages,” they therefore witnessed the crammed living conditions and the terror of deportation of their Jewish neighbours.^53^

Intermarried families like the Freibergers and *Hahns*, who, together with their children, were members of the Jewish community, were declared “non-privileged mixed marriages” and therefore subjected to a similar treatment as the general Jewish population. Their children, even though technically also “half-Jews” in Nazi terms, were classified *Geltungsjuden* according to the *Nuremberg Laws.* ^54^ Together with the Jewish population, they were gradually excluded from staple foods (milk, eggs, meat, wheat products)^55^ and evicted from their homes. While the *Hahns* left Vienna in a failed attempt to emigrate to Palestine via Yugoslavia only to return after a few months,^56^ the Freiberger family lost their apartment in December of 1939, because the caretaker of their building decided to give it to an “Aryan” family in the neighbourhood. They first moved to Moritz Freibergers sister Helene, who lived in an apartment in Grünentorgasse together with her daughter Elise and another Jewish sub-tenant. After witnessing the deportation of their relatives to Litzmannstadt (Łódż) in October of 1941,^57^ the Freibergers had to vacate the apartment and move to the Leopoldstadt, where the majority of the Jewish population was being concentrated. There they had to change apartments another two times. Lotte Freiberger described in an interview that her family basically lived from the food rations of her “Aryan” mother, since the provisions for her father and herself were severely limited.^58^ While being exposed to the same anti-Jewish legislation, “non-privileged mixed marriages” and their families, however, were deferred from deportation as long as the marriage with the “Aryan” spouse remained intact.^59^

## *Friends, Neighbours and Strangers*: Interactions of “Mixed Families” with their Jewish and Non-Jewish Environment

The *Anschluss* immediately disrupted social relationships between Jews and non-Jews. Lotte Freiberger, whose best friends were non*-*Jews, remembers distinctly how these girls suddenly made a point of ignoring her. She was subsequently excluded from high school and started taking occupational classes at the Jewish community, where she not only learned how to sew gloves, but also found new friends who were of similar background. On the weekends, Lotte and her new companions went to the Jewish part of the central cemetery, which had become the leisure place of Vienna’s Jewish population due to the fact that Jews were excluded from parks and recreation areas. There she also met *Judith Hahn*, who was among the younger youths gathering there. Together with other teenagers, Judith and Lotte played ball, helped with the cultivation and harvest of vegetables for the Jewish community or were hanging out with the boys, while forgetting their precarious situation for a short while.^60^

For the Baader family, the Nazi takeover did not only mean discrimination but also social isolation. While the Freiberger and *Hahn* families both benefitted from the infrastructure the Jewish community set up for their persecuted members, the Baaders were initially left without support. When in 1942 members of the *Hilfsstelle for “non-Aryan” Catholics* ^61^ stood at their door, Oskar Baader, known for his anti-clerical views, was caught by surprise. While he himself could not overcome his reservations towards the church, his son Gerhard greatly benefitted from the *Hilfsstelle’s* outreach. In the church youth group he was invited to he finally found a place where he was welcomed as a “youth among other youths.” There were, however, limits to this acceptance. When Gerhard fell in love with one of the girls during the preparation of a nativity play, the relationship was immediately prohibited by the parents of the girl, who forbade their daughter to closely socialize with “a Jew.”^62^

Ostracism also occurred within family circles. Some intermarried families faced reservations from their own relatives even before the war. After the Nazi takeover, they had to cope with the fact that non-Jewish relatives became party members and active combatants of the German Army, while their Jewish relatives were deported one by one.^63^ *Mathilde Hahn’s* Catholic family shunned her after her wedding to her Jewish husband and her conversion to Judaism. The fact that her twin brother Egon became an avid National Socialist shattered their relationship.^64^ Similarly, for the Freiberger family, the rift with non-Jewish family members in Sudetenland, who became active Nazi party members, proved irrevocable.^65^ Gerhard Baader, on the other hand, was in touch with his non*-*Jewish relatives even during the war, yet had to cope with his Catholic grandmother, who in her simple-mindedness was outspoken about her sympathies for the National Socialist regime.^66^

While *Geltungsjuden* had been immediately expelled from their schools and banned from any higher form of schooling, *Mischlinge* ^67^ like Gerhard Baader were still able to attend high school until 1942.^68^ In his case, this privilege had a distinct disadvantage, because he was known as the only “non-Aryan” at his school and had to endure daily discrimination. After his expulsion, he started to work as an unskilled labourer at a construction site, where he finally experienced acceptance and solidarity among his co-workers.^69^

The edict of 1 September 1941 obliging Jews to wear the stigmatizing yellow star was a turning point in relations between Jews and non-Jews. Along with the rest of the Jewish population, Jewish spouses of “non-privileged mixed marriages” as well as their children, who were classified as *Geltungsjuden*, were made visible as Jews, thereby becoming vulnerable to public assaults.^70^ Particularly members of younger generations regularly disregarded these Nazi regulations in order to attend places they were banned from as Jews.^71^ Eighteen-year-old Lotte Freiberger occasionally went to the cinema without the star together with her non-Jewish mother, but stopped doing so after recurrent reports of arrests and deportations of people who were caught.^72^ *Judith Hahn* on the other hand, who was in her early teens, did perhaps not fully realize the dangers and continued taking off the star on a regular basis. She was encouraged to do so by her father *Albert*, who also ventured out without the yellow star, while her “Aryan” mother was worried sick about them.^73^

The restrictive Nazi laws, heavily regulating Jewish daily life, occasionally also led to absurd situations for members of “mixed marriages” and their families. During a walk in the inner city, Lotte Freiberger and her non-Jewish mother passed the famous Demel pastry shop. Lotte longingly glanced at the display of cakes, but had been, as someone defined as Jewish, forbidden to buy and eat them since February of 1942.^74^ Her mother Mimi recognized her craving and went in to buy a cake. When she handed the package to her daughter, who was wearing the yellow star, she was apprehended by a Gestapo man, who had watched the scene and reprimanded her for forbidden “friendly relationships” towards Jews in public.^75^ After finding out the Jewish girl was in fact her daughter, he was taken aback and disappeared.^76^ In December of 1943, Mimi was apprehended another time for accompanying her Jewish husband Moritz, who as a Jew of a “non-privileged mixed marriage” was drafted to a forced labour assignment to shovel snow in the very early morning hours in front of the city court. Worried about her husband, who was physically challenged by a neuropathy resulting from his military service during World War One, she insisted on going with him. The presence of an “Aryan” woman accompanying a Jew marked by the yellow star enraged one of the guards. He had her arrested, but she was released after a few hours.^77^

For “mixed marriages” and their families, the relationships with their neighbours were crucial, since they shaped their daily lives. The Baader family, in spite of the fact they were considered privileged, were closely confronted with the fate of their Jewish neighbours, who lived in crammed apartments and were being deported one by one. Their son Gerhard was often woken up at night by the sound of boots on the stairs and the shouting that occurred as their neighbours were taken away.^78^ In addition, the daughter of their concierge, Leopoldine Frassl, closely monitored the remaining Jewish parties in the house. Through a staircase, she was able to watch the kitchen window of the Baader family. Cecilia Baader, who had a health damaging forced labour employment packaging poisonous substances, was able to get the permission of her supervisor to only work part-time. She therefore had to be careful not be seen by Frassl in the house and had to avoid entering the kitchen until the evening hours, since a denunciation could have led to her being deported for an alleged “unwillingness to work.” The fact that Leopoldine Frassl’s own brother Erwin was married to a Jewish woman, who happened to be Cecilia Baader’s sister Henriette, had no bearing on Leopoldine’s unshakeable fanaticism. A neighbour in the house, Otto Lauterbach, who was also protected through his marriage to a woman of Catholic decent, was less fortunate. He was denounced by Frassl for listening to foreign radio stations – a major violation of Nazi laws – and the “illegal wearing of a traditional Austrian folk suit *(Trachtenanzug),*” which was forbidden to Jews by anti-Jewish legislation. Subsequently, Otto Lauterbach was arrested and deported to Auschwitz. He survived and Leopoldine Frassl was put on trial after the war.^79^

The *Hahn* family, after returning from Yugoslavia, were also moved to the second district. There, they witnessed the disappearance of their Jewish neighbours as well. Their building hosted a temporary Jewish old-age home on the upper floor. In the summer of 1942, the thirteen-year-old *Judith Hahn* and her family observed the deportation of these helpless old people, who were pushed down the stairs under the gloating watch of their non-Jewish neighbours. *Judith* remembered one of these neighbours even inciting her hunting dog to bark at the fragile and frightened crowd huddled together on the open truck.^80^

There were, however, also acts of kindness and support among people in the neighbourhood and throughout the city. Sometimes these were small gestures such as a bowl of potatoes anonymously deposited at the doorstep or a package with a much-desired bologna sandwich that Lotte Freiberger was given secretly by a stranger, who was riding the tramway with her to work.^81^ In some cases, people even provided consistent support. A local non*-*Jewish grocery store owner in the neighbourhood of the *Hahn* family clandestinely supplied them with regular food provisions. Most of these groceries were used for the illegal activities of *Albert Hahn*, who was part of an Austrian resistance group that mainly supported people in hiding. With the help of his daughter *Judith*, he also sent food packages to acquaintances and friends, who in the meantime had been deported to Theresienstadt (Terezín).^82^ For those who remained in the Theresienstadt ghetto, these packages were a key factor in their survival.^83^

It was not only food that “mixed marriages” and their families were deprived of, but also normal caring human relationships with non*-*Jews that were rare, but meaningful if they occurred. According to an edict from 24 October 1941, “Aryans” were not allowed to have “friendly relationships” towards Jews in public.^84^ Lotte Freiberger, who was classified as *Geltungsjüdin*, was barred from regular leisure activities due to anti-Jewish legislation. One of her dreams as a teenager was to learn how to dance. Therefore, her mother approached Willibald Elmayer–Vestenbrugg (1885–1966), a cavalry captain of the military and founder of a prestigious dancing school. Elmayer instantly agreed to give Lotte private dance lessons and kept in touch with the family throughout the war.^85^

For the Baader family – in spite of their social isolation – there were nonetheless a few non*-*Jews who provided support. Members of the Catholic *Hilfsstelle* were regular visitors and even managed to gain the trust of Oskar Baader, his outspoken anti-clericalism notwithstanding. In addition, Baader’s closest friend continued to visit them in the second district. He always made a point of taking out the garbage for the family in his full *Wehrmacht* uniform, whenever he came to call on them – a sign of support that made a particular impression on the young Gerhard Baader. Finally, there was the wife of a local restaurant owner, Kuranda, who hired Oskar Baader as a tutor for her son Othmar. She immediately became aware of the difficult situation the Baaders faced and convinced her husband to welcome Gerhard Baader as a companion to their son into their home. They even invited him to come along for family outings in the country side, which Gerhard particularly enjoyed, since members of “mixed families” were already restricted from moving around freely outside of the city limits. These visits only came to a halt when Gerhard, in a carefree moment, went on a hiking trip to the Rax mountain area with a friend of Othmar, who had no clue about Gerhard’s “mixed” background. On the way back, they were apprehended by military police near Wiener Neustadt, where Gerhard was arrested and sent back to Vienna, while his companion was reprimanded for socializing with “a Jew.” After learning about this incident, Vinzenz Kuranda decided that as a local restaurant owner he could no longer afford to openly invite Gerhard into his home. Nevertheless, his wife still continued to support the Baader family with much-welcomed groceries, which Gerhard clandestinely picked up every weekend from their storage. These additional foods also provided the Baaders with the opportunity to send parcels with groceries to Cecilia Baader’s brother Julius Adler in Theresienstadt. Adler was able to survive the war there together with his wife Rosa.^86^

While acts of kindness or support are mentioned in autobiographical accounts and interviews, they are nevertheless presented as an exception in an environment that had suddenly turned hostile. The traumatic experiences following the *Anschluss* to Nazi Germany in March 1938 and the humiliation and the sudden loss of rights that followed were too deeply imprinted. Overnight, neighbours and friends had turned against them, and denunciation continued to be a constant threat.

## *Precarious Protection* – The Crucial Role of the “Aryan” Parent or Spouse

Existing marriages between Jews and non-Jews, as well as the presence of their “half-Jewish” children, presented a permanent threat to the integrity of the Nazi regime. In conjunction with the so-called Final Solution, this “unsolved problem” played an important role in the discussions during the infamous Wannsee Conference in January 1942 and its follow-up meetings in March and October of the same year. Internal differences within the Nazi party and concerns that “Aryan” family members would cause public unrest ultimately spared this group from the full force of the radical measures applied to the rest of the Jewish population, even if plans for the ultimate inclusion of “half-Jews” and Jewish partners of “mixed marriages” in the Final Solution were never abandoned.^87^

In the course of the mass deportations between February 1941 and October 1942, the majority of the Austrian Jewish population was deported from Vienna.^88^ Jewish spouses of “mixed marriages” as well as their children were officially deferred from these transports, as long as the matrimony remained valid. If it was dissolved due to divorce or the death of the “Aryan” spouse, this protection ended immediately, unless there were under-aged children classified as *Mischlinge.* ^89^ At the same time, Jewish spouses, as well as *Geltungsjuden* coming of age, lost the protection of their “Aryan” parent or partner, if they no longer shared the same household.^90^ Non*-*Jewish spouses of “non-privileged mixed marriages,” who could no longer endure the conditions in crowded Jewish apartments, thereby often unknowingly endangered their Jewish spouses, if they moved out.^91^

Lotte Freiberger turned nineteen in the summer of 1942, at the peak of the mass deportations from Vienna. Around this time, she was summoned for deportation for the first time. When the SS came to pick her up in the family apartment, she was ready to jump out of the window and was only prevented from doing so by her father. They came back on two more occasions, always unannounced. Each time Lotte was told to pack, while the SS had her papers checked, and each time she ended up being excluded from the transport – after hours of nerve-wracking uncertainty. Even though the SS came only for Lotte, who had officially entered adulthood, her mother decided that the family was not to be torn apart and all three ended up packing, until they were given the all-clear that they were not being deported. While Lotte Freiberger was ultimately able to remain in Vienna because she shared the same household with her “Aryan” mother, she nevertheless was made aware of the very fragile nature of her protection.^92^ Since deportation guidelines were never published, as they were considered “*Geheime Reichssache,”* members of “mixed marriages” and their families did not officially learn about the key factors of their safeguarding. Instead, they were left to their own instincts on how to uphold their protection.^93^

While in Vienna, the mass deportations of the majority of the Jewish population were already concluded by the autumn of 1942, they were still under way in Berlin.^94^ In fact, it was the infamous *Fabrik Aktion* in February of 1943 that was the prelude to the final deportation of Berlin’s Jewish population, regardless of their value for the work force. However, while Jewish partners of “mixed marriages” were arrested in the course of this *Aktion* as well, it was – as Wolf Gruner has shown in great detail – never the intention to deport all of them. Nevertheless, their papers were checked whether their protection through marriage was still valid. The public protest of their “Aryan” spouses outside of the Rosenstraße collection camp – regardless of its importance as a public sign of resistance – was therefore not the reason for their release, as previously assumed.^95^

During the last years of the war and after the deportation of the majority of the Jewish population, the remaining Jews came under increased scrutiny, and even trivial infractions against Nazi laws could lead to imprisonment and deportation. In the autumn of 1943, the Gestapo arrested eighteen-year-old Friedrich Braun, classified as a *Geltungsjude*, who lived with his “Aryan” mother and stepfather, for his “absence from work.” He was deported to Auschwitz, where he was murdered in January 1944.^96^ Even activities like going to the cinema could be fatal, since it was forbidden to Jews, and therefore one could only enter if one removed the yellow star, which was itself a major offense. In 1943, the twenty-one-year-old Katharina Fischer was arrested for “disregarding the mandatory labelling” – the yellow star she was supposed to wear as *Geltungsjüdin*. She was deported to Auschwitz and killed in December of that year.^97^ Consequently, these wide-ranging stipulations led to a criminalization of normal, daily activities, since survival strategies, such as getting extra food from the black market to supplement the meagre food rations, were dangerous. This can be seen in the increased number of arrests quoted in the Gestapo daily reports during the final years of the war.^98^ Regardless of the dangers involved, some of the remaining Jews, particularly younger generations, continued to take off the yellow star. Apart from the comprehensible motivation to have moments of “normal life,” this was also an act of self-assertion and agency. *Judith Hahn* ^99^ reflected on this in an interview:

If one talks about this today, this all sounds downright silly, because actually we were taking a risk for nothing, which we did not realize then. We did it. And maybe it also contributed to the fact: We felt stronger, if we did not show fear or if we were carefree. One was able to endure it better.^100^

In the autumn of 1944, even “privileged families” were increasingly affected by the radicalization of the Nazi regime. Male *Mischlinge* ^101^ as well as “Aryan” spouses of “mixed marriages,” who both were considered “unworthy” of military service, were drafted for forced labour deployment in the OT.^102^ There are indications that this was intended to disrupt these families and to impair the protection of “Aryan” spouses in order to facilitate future deportations.^103^ Gerhard Baader, who was drafted as a sixteen-year old, was already aware of the imminent defeat of the German Army. Together with his fellow inmates, most of them teenage *Mischlinge* ^104^ like himself, he used every opportunity to evade orders during his OT deployment and was not even afraid to commit minor infringements, such as sneaking out at night to the nearby Prater amusement park. Older generations like the one to which his father belonged had a much harder time, as they tended to wear themselves out by following orders to the letter. Oskar Baader was sent to a forced labour unit on the border of Hungary, designated to build the South-East-Wall *(Südostwall)*, where he was placed under the command of former criminals. As a veteran officer of World War One, the demeaning treatment he experienced was the ultimate humiliation he never recovered from.^105^

Survival was not guaranteed until the end. In January 1945, only months before the end of the war, the *Reichssicherheitshauptamt* (Reich Main Security Office, RSHA) finally issued an edict ordering the deportation of *Geltungsjuden* and Jewish spouses of “mixed marriages” to Theresienstadt.^106^ While in Germany about 1,600 persons were deported from a number of cities by the end of February, local Nazi officials in Vienna cancelled the transport planned for 26 February 1945 because of the approaching front.^107^

## Postwar Lives and Identities

After the war, intermarried families were considered by survivors as those to whom “nothing had happened,” since they had been able to remain in Vienna, seemingly unscathed, while everyone else was being deported. This certainly had an impact on the self-definition of intermarried families. While learning the gruesome facts of the Holocaust, their own experience of discrimination and persecution suddenly seemed insignificant by comparison. In addition, the *Hahn* and Baader family had to cope with the loss of their “Aryan” spouse and parent, who had been the guarantee of their survival during the war years. While the reason for these suicides may never be fully comprehended, it nevertheless indicates, that these war time experiences took their toll on intermarried families.

Children of intermarried families were acknowledged as victims of National Socialism rather late in Austria. For those, who as *Mischlinge* did not have to wear the yellow star, recognition only came the late 1990s.^108^ Therefore, their status of being “in-between” often continued through their postwar lives, since they were neither included in organizations of Nazi victims nor did they fully belong to general Austrian society, considering the fact that their narratives of wartime differed significantly from non-Jewish Austrians. Gerhard Baader found a new identity in Socialism. Nevertheless, he described his first awakening to his Jewish identity at the funeral of his Jewish grandmother in 1942. Due to the lack of Jewish men in the wake of ongoing mass deportations, Gerhard was counted in the *minyan* for the first time, when the *Kaddish* was recited. In spite of the fact that during this time he was an active member of the local Catholic church youth group and regularly attended Sunday masses, this nevertheless made a lasting impression on him. After the war, he loosened ties with the church, which – due to the dauntless sermons of the charismatic local priest Arnold Dolezal (1902–1978) – had also functioned for him as a forum to express his opposition to the Nazi regime. He immediately started to engage himself in Social Democratic youth and student organizations and became a renowned medical historian. In his later life, however, he reconnected with his Jewish roots and today he is an active senior board member of the Oranienburg synagogue in Berlin.^109^ Lotte Freiberger, on the other hand, who narrowly escaped deportation, distanced herself from the Jewish community after the war. Her marriage to a Christian was one of her attempts to blend into Austrian society – an attempt that was bound to fail, as was her marriage. In an interview, she revealed that it took her many years to become comfortable to visit the second district again, where she had survived the war years. After her retirement, however, she became a volunteer with the Documentation Centre of Austrian Resistance (DÖW), which brought her closer to her Jewish roots again. Nevertheless, she is still cautious about her Jewish background and only gives interviews under her maiden name Freiberger in order to “protect her daughter,” who was raised Catholic.^110^ *Judith Hahn*, finally, had already been raised with a strong sense of Jewish identity. The Nazi racist laws therefore did not fundamentally change the way she perceived herself, in spite of their discriminatory character. After the war, she immersed herself in her studies in order to catch up with the education she had been barred from during the war years. *Judith* and her friend Helga, a survivor of Theresienstadt camp, made a point of excelling at every exam, in order to show their non-Jewish classmates that they were able to so. They had no intention of blending in and proudly carried a necklace with the star of David in class. *Judith* also immediately got in touch with the Zionist youth movement *Hakoah*, where she met her husband, a Hungarian Holocaust survivor. Their plans to make *Aliyah* to Israel, however, did not materialize and they remained in Vienna.^111^ As with most children of intermarried families, the postwar lives of Gerhard Baader, Lotte Freiberger and *Judith Hahn* took very different paths. While Gerhard Baader as an active Social Democrat became involved in Jewish community life in recent years, for Lotte Freiberger the defining moment in regard to her Jewish descent remained that of caution – quite in contrast to *Judith Hahn*, whose strong sense of Jewish identity seemed hardly affected by the Nazi years. Nevertheless, the fact that the descendants of the *Hahn* family requested the name to be anonymized in order not to endanger their place in today’s Jewish community by highlighting the Catholic descent of one of their maternal ancestors indicates that questions of identity and belonging for these families remain crucial issues until today.

## Conclusion

Intermarried families were under enormous strain during the years of the Nazi regime. Particularly after the end of mass deportations, the remaining Jewish population came under increased scrutiny by the authorities. Since members of “mixed marriages” and their families did not officially learn about the key factors of their safeguarding, they were left to their own instincts on how to uphold their protection. In addition, their relationship with their non-Jewish environment was greatly affected from the start. While many of their friends and neighbours immediately turned against them after the Nazi takeover, there were also a few who provided support, even if only in small gestures. In the course of this paper, the complexities of these relationships have been outlined. Wrongdoers were usually ordinary people, who were often not even party members: caretakers, who offered the apartment of Jewish families in their building to “Aryans”; neighbours, who incited their hunting dog at helpless Jews; or, in the case of the Baaders it could even be a woman related by marriage, who was ready to report them at any given time. Support, on the other hand, could come from unexpected sides such as former business competitors in the case of Moritz Freiberger, a *Wehrmacht* soldier, who upheld his friendship with his former colleague Oskar Baader or clandestine dance classes given by a cavalry captain of the military.

It is the microhistorical lens that reveals the simultaneity of the non-simultaneous: The Baader family, while theoretically being classified as privileged by the NS-regime, was nevertheless exposed to similar persecution measures as the *Hahn* and Freiberger families. At the same time, the way they dealt with Nazi oppression differed to some degree. While the Jewish members of the *Hahn* family were still able to take pride in their Jewish identity and were overall motivated to withstand the regime, the Baader and Freiberger families instead acted with great caution. There were, however, generational differences, since Gerhard Baader was eager to participate in activities that other non*-*Jewish teenagers were engaged in and was not afraid to risk minor infringements, much to the distress of his parents. Interestingly, it was his forced labour assignment that sparked this youthful nonchalance, since he found unexpected comradeship with his fellow construction workers, who took him along. While the Baader, Freiberger and *Hahn* family lived in close proximity to one another in the second district of Vienna, where the remaining Jewish population was being concentrated, they hardly formed bonds with each other nor with many other “mixed marriages” and their families. When “non-privileged families” shared living quarters with other families, nerves were often “strained at utmost,” as Lotte Freiberger remembered, since all were facing an uncertain future. “Cooking took place in a small kitchen. There were daily arguments and bickering, everyone’s nerves were on edge, living together became an ordeal,” she recalled.^112^ Consequently, people living in crammed conditions did not necessarily form bonds of solidarity with each other. It was the younger generation that managed to form “emotional communities” of their own – Gerhard Baader with his fellow construction workers and in the church youth group and *Judith Hahn* and Lotte Freiberger with other youths at the Jewish cemetery, their playground. These bonds helped them cope with discrimination and persecution and sometimes even made them forget the dire reality for a short while.

Intermarried families, who – by definition – were navigating between Jewish and non-Jewish worlds, did usually not fully belong to any side. While this status of being “in-between” granted them a wider room of manoeuvre than the general Jewish population, it also affected their identity and sense of belonging. The fact that they were as “non-Aryans” excluded from society did not necessarily lead to full acceptance from the Jewish side, particularly if they had already stepped out of the Jewish community before. Nor did it immediately create a desire to belong. This was particularly true for the Baaders, who kept their distance from the Jewish community, while being excluded from society at large. Their social isolation was therefore also a result of this conflict. At the same time, their restraint in their social contacts had already started before the *Anschluss*, since they were supporters of the forbidden Social Democrats under the Austrofascist regime. The Freibergers, who were officially members of the Jewish community, had become estranged from Judaism due to reservations from Jewish family members against their marriage. While, after the *Anschluss*, their daughter Lotte benefitted from the infrastructure of the Jewish community for their persecuted members, Moritz and Mimi Freiberger led secluded lives with only a few social contacts. It was only the *Hahn* family, whose identity seemed hardly disrupted, since they were already proudly identifying Jews before the war. Nonetheless, not all members of the family were able to endure discrimination and persecution with persistence and pride. *Mathilde Hahn* not only suffered from the degradation they were subjected to, but also from ostracism by her own family, who became ardent supporters of the National Socialist regime.

As previously demonstrated, the living conditions of members of “mixed marriages” and their families changed over time. Particularly during the last years of the war their protection became more precarious, since even trivial infractions against Nazi laws could lead to imprisonment and deportation. The pervasive feeling for “mixed families” therefore was one of high alert and a mandatory lack of trust in their day-to-day encounters with their non*-*Jewish environment, since anyone could potentially turn against or even denounce them. During this time, the presence of the “Aryan” family member was essential for their protection and survival. The enormous strain on intermarried families took its toll. The postwar losses of the Baader and *Hahn* families can be also attributed to the years of persecution.

## ORCID

*Michaela Raggam-Blesch* http://orcid.org/0000-0002-7476-5220

## Footnotes

1 Name anonymized on request of the family.

2 Shoah Foundation interview, Visual History Archive (VHA), Interview Code 38836.

3 Gerhard Baader, interview by the author on 2 July 2012 in Vienna.

4 In the context of this paper, the term “intermarried families” refers to marriages between people of Jewish and non*-*Jewish descent together with their children, while the term “mixed marriage” as literal translation of the German word *Mischehe* indicates Nazi terminology.

5 Barbara Rosenwein, “Worrying about Emotions in History,” *American Historical Review* 107, no. 3 (2002): 821–45.

6 Research indicates, that most “mixed families” coped with discrimination and persecution alone. James F. Tent, *In the Shadow of the Holocaust: Nazi Persecution of Jewish-Christian Germans* (Lawrence: University Press of Kansas, 2003), 15–16.

7 As with most oral history interviews, these were interviews with survivors, who were children and young adults during the Nazi regime. The collection of Shoah Foundation interviews at the Visual History Archive (VHA) contains comparatively few interviews with individuals who survived as members of “mixed families.” This might also be linked with the fact that the fate of these families during the Nazi regime has only recently gained more attention in Holocaust studies.

8 The children of the Baader and Freiberger family were interviewed by myself multiple times in the course of my research project on children of intermarried families in Vienna during the Nazi regime. In the case of the *Hahn* family, the Shoah Foundation interview with their daughter, who already passed away, is the only available source. The families were chosen from a data base of interviews with thirty-eight descendants of intermarried families conducted by myself, fourteen Shoah Foundation interviews and eighteen interview transcripts at the Documentation Centre of Austrian Resistance (DÖW). The autobiographical manuscript written by the daughter of the Freiberger family is also in the holdings of the DÖW.

9 On Oral History and Holocaust testimonies: Boaz Cohen, “Holocaust Testimonies and Historical Writing: Debates, Innovations, and Problems in the Early Postwar Period,” *Yad Vashem Studies* 75, no. 2 (2017): 159–83; Sharon Kangisser Cohen, *Testimony and Time: Holocaust Survivors Remember* (Jerusalem: Yad Vashem, 2014); Ulrike Jureit, *Erinnerungsmuster: Zur Methodik lebensgeschichtlicher Interviews mit Überlebenden* (Hamburg: Ergebnisse Verlag, 1999); Lawrence Langer, *Holocaust Testimonies: The Ruins of Memory* (New Haven: Yale University Press, 1991); Alexander von Plato, “Zeitzeugen und die historische Zukunft. Erinnerung, kommunikative Tradierung und kollektives Gedächtnis in der quantitativen Geschichtswissenschaft - ein Problemaufriss,” *BIOS* , no. 13 (2000): 5–28; James Edward Young, *Writing and Rewriting the Holocaust: Narrative and the Consequences of Interpretation* (Bloomington: Indiana University Press, 1990).

10 See for example Doron Rabinovici, “‘Jidn, sogt, wer schtejt bajm tojer?’: Der Fall Franz Murer – ein österreichischer Schauprozeß gegen die Opfer,” in *Ess firt kejn weg zurik … Geschichte und Lieder des Ghettos von Wilna 1941–1943*, ed. Florian Freund, Franz Ruttner, and Hans Safrian (Vienna: Picus, 1992), 91–122.

11 Gabriele Rosenthal, “Über die Zuverlässigkeit autobiographischer Texte,” in *Den Holocaust erzählen: Historiographie zwischen wissenschaftlicher Empirie und narrative Kreativität*, ed. Norbert Frei and Wulf Kansteiner (Göttingen: Wallstein, 2013), 165–72; Christopher Browning, *Remembering Survival: Inside a Nazi Slave-labor Camp* (New York: W. W. Norton & Company, 2010), 9.

12 Saul Friedländer, *Den Holocaust beschreiben: Auf dem Weg zu einer integrierten Geschichte* (Göttingen: Wallstein, 2007), 7–27.

13 Claire Zalc and Tal Bruttmann, eds., *Microhistories of the Holocaust* (Oxford and New York: Berghahn, 2017), 1–13. See also Sigurður Gylfi Magnússon and István M. Szijártó, *What Is Microhistory? Theory and Practice* (New York: Routledge, 2013), 4–5.

14 According to the national census in May of 1939, Vienna was the city with the highest Jewish population within Nazi Germany: Statistik des Deutschen Reichs, “Die Juden und jüdische Mischlinge im Deutschen Reich,” in *Die Bevölkerung des Deutschen Reichs nach den Ergebnissen der Volkszählung 1939*, vol. 552/4 (Berlin: Paul Schmidt, 1944), 6–8. Even before the Nazi takeover in 1938, ninety-two per cent of the Austrian Jewish population lived in Vienna: Jonny Moser, *Demographie der jüdischen Bevölkerung Österreichs 1938–1945* (Vienna: Dokumentationsarchiv des österreichischen Widerstandes, 1999), 9, 16.

15 The term “mixed marriage” as literal translation of the German word *Mischehe* indicates Nazi terminology. See footnote 4.

16 Intermarried families in other countries under Nazi control were treated differently. While in many Western European countries, “mixed marriages” were at least initially exempt from deportations, Jewish members of intermarried families in occupied eastern territories such as Poland could never rely on any protection. H.G. Adler, *Der verwaltete Mensch: Studien zur Deportation der Juden aus Deutschland* (Tübingen: Mohr, 1974), 283*–*4; *Enzyklopädie des Holocaust: Die Verfolgung und Ermordung der europäischen Juden*, ed. Israel Gutman, Eberhard Jäckel, Peter Longerich, and Julius H. Schoeps, vol. 2 (Munich and Zurich: Piper, 1995), 956*–*8; Wolf Gruner, *Widerstand in der Rosenstraße: Die Fabrik*–*Aktion und die Verfolgung der “Mischehen” 1943* (Frankfurt am Main: Fischer TB Verlag, 2005), 96*–*7.

17 Monica Kingreen, “Tödliche Verfolgung von als jüdisch klassifizierten ‘Mischehepartnern’ in der Rhein-Main-Region,” in *Der halbe Stern: Verfolgungsgeschichte und Identitätsproblematik von Personen und Familien teiljüdischer Herkunft*, ed. Brigitte Gensch and Sonja Grabowsky (Gießen: Haland & Wirth, 2010), 57*–*66. For the situation in Munich, see Max Strnad, “The Fortune of Survival – Intermarried German Jews in the Dying Breath of the ‘Thousand-Year Reich’,” *Dapim: Studies on the Holocaust* 29, no. 3 (2015): 173–96. On Berlin, see Maria von der Heydt, “‘Geltungsjuden’ und jüdische Gemeinde in Berlin 1939–1945,” *Zeitgeschichte* 43, no. 5 (2016): 308–23.

18 Individuals with one Jewish and one non*-*Jewish parent, who were baptized or without denomination at the time of the introduction of the *Nuremberg Laws*. See footnote 49.

19 Raul Hilberg, *Die Vernichtung der europäischen Juden*, vol. 2, 10th ed. (Frankfurt am Main: Fischer TB Verlag, 2007), 436; H.G. Adler, *Das Schicksal der jüdischen “Mischlinge” im Zweiten Weltkrieg* (Stuttgart: Süddeutschen Rundfunk, 1966); Hermann Graml, “Zur Stellung der Mischlinge 1. Grades,” in *Gutachten des Instituts für Zeitgeschichte*, vol. 2 (Munich: Institut für Zeitgeschichte, 1966).

20 Ursula Büttner, *Die Not der Juden teilen: Christlich-jüdische Familien im Dritten Reich* (Hamburg: Christians, 1988); Jeremy Noakes, “The Development of Nazi Policy towards the German*-*Jewish ‘Mischlinge’ 1933–1945,” *LBI Year Book* 34 (1989): 291*–*354; Beate Meyer, *“Jüdische Mischlinge”: Rassenpolitik und Verfolgungserfahrung 1933–1945* (Hamburg: Dölling und Galitz, 1999); Tent, *In the Shadow of the Holocaust*.

21 Victor Klemperer, *Ich will Zeugnis abgeben bis zum letzten: Tagebücher 1942–1945* (Berlin: Aufbau, 1995).

22 Till van Rahden, “Weder Milieu noch Konfession: Die situative Ethnizität der deutschen Juden im Kaiserreich in vergleichender Perspektive,” in *Religion im Kaiserreich. Milieus – Mentalitäten – Krisen*, ed. Olaf Blaschke and Frank-Michael Kuhlemann (Gütersloh: Kaiser Gütersloher Verlagshaus, 1996), 409–34; Lisa Silverman, *Becoming Austrians: Jews and Culture between the World Wars* (Oxford: Oxford University Press, 2012).

23 The term *Anschluss* refers to the Nazi takeover of Austria in March 1938.

24 Bruce F. Pauley, *From Prejudice to Persecution: A History of Austrian Anti-Semitism* (Chapel Hill: University of North Carolina Press, 1992); Klaus Taschwer, *Hochburg des Antisemitismus: Der Niedergang der Universität Wien im 20. Jahrhundert* (Vienna: Czernin, 2015); Robert S. Wistrich, *Die Juden Wiens im Zeitalter Kaiser Franz Josephs* (Vienna: Böhlau, 1999), 171–97.

25 Both Cecilia and Oskar Baader were students of English literature. Universitätsarchiv Wien (UAW), Philosophische Fakultät, Nationale 1924/25, Cecilia Adler; UAW, Philosophische Fakultät, Nationale 1920/21, Oskar Baader.

26 The DAA only accepted “Aryans” as its members. Roman Pils, “‘Ein Gelehrter ist kein Politiker:’ Die Professoren der Wiener Anglistik im Kontext des Nationalsozialismus,” in *Geisteswissenschaften im Nationalsozialismus: Das Beispiel der Universität Wien*, ed. Mitchell G. Ash, Wolfram Nieß and Ramon Pils (Göttingen: V&R unipress, 2010), 455–86.

27 Baader, Interview, 2012; telephone interview with Gerhard Baader by the author on 30 May 2017. For more on Austrofascism, see most recently: Florian Wenninger and Lucile Dreidemy, eds., *Das Dollfuß-Schuschnigg*-*Regime 1933*–*1938: Vermessung eines Forschungsfeldes* (Vienna: Böhlau, 2013); Emmerich Tálos, *Das austrofaschistische Österreich 1933–1938* (Vienna: Lit Verlag, 2017).

28 Name anonymized on request of the family.

29 Pauley, *From Prejudice to Persecution*, 132–40; Taschwer, *Hochburg des Antisemitismus*, 70–97; Wistrich, *Die Juden Wiens*, 179–81.

30 Wistrich, *Die Juden Wiens*, 287–312.

31 Name anonymized on request of the family.

32 Name anonymized on request of the family.

33 The school was named after its founder, the Jewish philanthropist and educator Eugenie Schwarzwald (1872–1940 Zurich), who was a reformer of girl’s education in Austria.

34 Universitätsarchiv Wien (UAW), Juristen Nationale 1924/25; VHA Interview (38836), 1997.

35 Michaela Raggam-Blesch, “Alltag unter prekärem Schutz: ‘Mischlinge’ und ‘Geltungsjuden’ im NS-Regime in Wien,” *Zeitgeschichte* 43, no. 5 (2016): 293, 295.

36 Lotte Freiberger (maiden name), interview by the author on 30 April 2009 and 26 January 2017 in Vienna.

37 Freiberger, Interview, 2009; Freiberger, Interview, 2017.

38 The slogans of the Austrofascist regime were campaigning for the ballot that was cancelled due to an ultimatum by the German regime and the invasion of German troops. Dieter J. Hecht, Eleonore Lappin-Eppel, and Michaela Raggam-Blesch, *Topographie der Shoah: Gedächtnisorte des zerstörten jüdischen Wien*, 2nd ed. (Vienna: Mandelbaum, 2018), 16*–*41.

39 Doron Rabinovici, *Instanzen der Ohnmacht: Wien 1938*–*1945. Der Weg zum Judenrat* (Frankfurt am Main: Jüdischer Verlag, 2000), 57*–*60, 124; Saul Friedländer, *Das Dritte Reich und die Juden, Gesamtausgabe* (Munich: DTV, 2008), 262*–*9; Hecht, Lappin-Eppel, and Raggam-Blesch, *Topographie*, 16*–*17.

40 Freiberger, Interview, 2017; Baader, Interview, 2012.

41 Joseph Walk, ed., *Das Sonderrecht für die Juden im NS-Staat: Eine Sammlung der gesetzlichen Maßnahmen und Richtlinien – Inhalt und Bedeutung* (Heidelberg: Müller, 1981), 127. *Gesetzblatt für das Land Österreich* (27 May 1938), 421.

42 Noakes, “Development of Nazi Policy,” 291*–*354; Hilberg, *Vernichtung*, 436*–*45; Friedländer, *Das Dritte Reich und die Juden*, 164–8, 721*–*5.

43 Gruner, *Rosenstraße*, 178–81; Meyer, *“Jüdische Mischlinge”*, 91*–*4, 185*–*90; Evan Burr Bukey, *Jews and Intermarriage in Nazi Austria* (New York: Cambridge University Press, 2011), 88*–*94.

44 Baader, Interview, 2012.

45 The “Brown House” in Hirschengasse 25 was the headquarters of the illegal Nazi party in Vienna. Today it is a guest house for Austrian students visiting Vienna. Only in the course of a research project at the Vienna University in 2009 was the Nazi past of the building brought to light. https://ahh.univie.ac.at (accessed 2 June 2018).

46 Freiberger, Interview, 2009; Freiberger, Interview, 2017.

47 While *Mathilde Hahn* worked as a sales representative for books, Mimi Freiberger sold products her daughter Lotte handcrafted in occupational classes. Later on, Lotte and her father’s forced labour employment provided the family with an income. VHA Interview (38836), 1997; Freiberger, Interview, 2017.

48 The distinction between “privileged” and “non-privileged mixed marriages” was introduced shortly after the November pogrom and the surge of anti-Jewish legislation. The privileged group was thereby exempt from some of the anti-Jewish laws and taxes. Noakes, “Development of Nazi Policy,” 337.

49 The First Supplementary Decree of the *Nuremberg Laws*, issued on 14 November 1935, defined people with a Jewish and an “Aryan” parent either as so-called *Mischlinge of the first degree* or as *Geltungsjuden* – depending on their denomination. “Half-Jews,” who were baptized or without denomination, were defined *Mischlinge*. They were neither considered “Aryan” nor Jewish and literally personified the status of being “in between.” The *Nuremberg Laws* also classified individuals with only one Jewish grandparent as *Mischlinge of the second degree (Quarter-Jews)*, who were usually less targeted. In this paper, the term *Mischling* will only refer to *Mischlinge of the first* degree. Walk, *Sonderrecht*, 127, 139*–*40, 233, 379.

50 Ibid., 292.

51 In Austria, the law protecting tenants was only valid for buildings built before 1917, which excluded tenants in the community buildings built by the Social Democrats in the 1920s. Hecht, Lappin-Eppel, and Raggam-Blesch, *Topographie*, 49*–*52.

52 The term *Judenhaus* refers to a building that mainly hosted apartments with Jewish inhabitants, who had been moved there not by their own choice. Friedländer, *Das Dritte Reich*, 313*–*4; Hecht, Lappin-Eppel, and Raggam-Blesch, *Topographie*, 395*–*409.

53 Baader, Interview, 2012; Baader, Telephone Interview, 2017. Offical letter from the Vienna municipality to the district court of Hietzing on 10 December 1938, terminating the tenancy agreement with Dr. Oskar Baader for the end of the month, holdings of Gerhard Baader.

54 Individuals with one Jewish and one non-Jewish parent, who were registered with the Jewish Community (IKG) at the time of the introduction of the Nuremberg Laws in 1935, were considered *Geltungsjuden* and subjected to the same discriminatory regulations as the rest of the Jewish population. Walk, *Sonderrecht*, 127, 139*–*40, 347.

55 Walk, *Sonderrecht*, 312, 318–9, 346, 378, 380.

56 VHA Interview (38836), 1997.

57 Helene and Elise Freyberger were deported to Litzmannstadt (Łódż) on 15 October 1941 and ultimately murdered. Of the 5,000 Viennese Jews deported there, only 34 survivors are known. Most of them were killed in Chełmno (Kulmhof) extermination camp. Bertrand Perz, “Viennese Jews in Litzmannstadt Ghetto,” in *Post 41: Berichte aus dem Getto Litzmannstadt / Reports from Litzmannstadt Ghetto. Ein Gedenkbuch / A Memorial Book*, ed. Angelika Brechelmacher, Bertrand Perz, and Regina Wonisch (Vienna: Mandelbaum, 2015), 19–69. For more on the conditions in the ghetto Litzmannstadt, see Andrea Löw, *Juden im Getto Litzmannstadt: Lebensbedingungen, Selbstwahrnehmung, Verhalten* (Göttingen: Wallstein, 2006). Database of Austrian Shoah Victims (DBSHOA): http://www.doew.at/erinnern/personendatenbanken/shoah-opfer (accessed 3 June 2019).

58 Freiberger, Interview, 2009; Walk, *Sonderrecht*, 346, 378, 380, 387.

59 Gruner, *Rosenstraße*, 50*–*2. Deportation guidelines of 11 October 1941 and 31 January 1942 in: Alfred Gottwaldt and Diana Schulle, *Die “Judendeportationen” aus dem Deutschen Reich 1941–1945: Eine kommentierte Chronologie* (Wiesbaden: Marixverlag, 2005), 56–8, 140–4.

60 Freiberger, Interview, 2017; VHA Interview (38836), 1997; Elisabeth W. Trahan, *Walking with Ghosts: A Jewish Childhood in Wartime Vienna* (New York: Peter Lang, 1998), 146–84. See also: Michaela Raggam-Blesch, “Survival of a Peculiar Remnant: The Jewish Population of Vienna during the Last Years of the War,” *Dapim: Studies on the Holocaust* 29, no. 3 (2015): 207*–*9.

61 For more on the *Hilfsstelle* and their aid for persecuted Catholic “non-Aryans,” see: Hecht, Lappin-Eppel, and Raggam-Blesch, *Topographie*, 339–48.

62 Baader, Interview, 2012. In fact, the discriminatory Nazi laws also limited the legal choices of partners for children of “mixed marriages” (“half-Jews”). While *Geltungsjuden* were forbidden to have relationships with “Aryans,” *Mischlinge* like Gerhard Baader needed permission to marry but were not officially banned from relationships until May and July 1942. Nevertheless, they were subject to frequent denunciations. Walk, *Sonderrecht*, 139–40, 372, 382; Noakes, “Development of Nazi Policy,” 310*–*3. See also: Raggam-Blesch, “Alltag unter prekärem Schutz,” 298*–*9.

63 Raggam-Blesch, “Alltag unter prekärem Schutz,” 292*–*307.

64 Telephone interview with the daughter of *Judith Hahn* (name anonymized), 20 October 2017.

65 Freiberger, Interview, 2017.

66 Baader, Interview, 2012.

67 In the course of this paper, the term *Mischling* is only used for children of a Jewish and a non*-*Jewish parent *(Mischling of the first degree*). See footnote 49.

68 Walk, *Sonderrecht*, 379; Hecht, Lappin-Eppel, and Raggam-Blesch, *Topographie*, 101*–*4.

69 Baader, Interview, 2012.

70 Walk, *Sonderrecht*, 347–8.

71 Raggam-Blesch, “Alltag unter prekärem Schutz,” 295*–*6.

72 Freiberger, Interview, 2009.

73 VHA Interview (38836), 1997.

74 Walk, *Sonderrecht*, 363.

75 Ibid., 353.

76 Freiberger, Interview, 2009.

77 Ibid.; Freiberger, Interview, 2017.

78 Baader, Interview, 2012.

79 Ibid.; “Tagesbericht der Gestapo Wien,” USHMM (United States Holocaust Memorial Museum) RG–17.035, TB 3 (7*–*9 December 1943), 5; Oberösterreichisches Landesarchiv, LG Linz, Vg 8 Vr 6477/47.

80 VHA Interview (38836), 1997; Hecht, Lappin-Eppel, and Raggam-Blesch, *Topographie*, 252.

81 Freiberger, Interview, 2009; Freiberger, Interview, 2017.

82 VHA Interview (38836), 1997.

83 Many of the deportees to Theresienstadt were later on deported to extermination camps and sites of mass killings such as Auschwitz-Birkenau, Sobibór, Treblinka and Maly Trostinec. Those who remained were exposed to dire sanitary conditions and malnutrition. For more on the conditions in the Theresienstadt ghetto, see H.G. Adler, *Theresienstadt 1941–1945: Das Antlitz einer Zwangsgemeinschaft* (Tübingen: Mohr, 1960); Wolfgang Benz, *Theresienstadt: Eine Geschichte von Täuschung und Vernichtung* (Munich: Beck, 2013); Anna Hájková, “Mutmaßungen über deutsche Juden: Alte Menschen aus Deutschland im Theresienstädter Ghetto,“ in *Alltag im Holocaust. Jüdisches Leben im Großdeutschen Reich 1941–1945*, ed. Doris Bergen, Anna Hájková, and Andrea Löw (Munich: Oldenbourg, 2013), 179–98.

84 Walk, *Sonderrecht*, 353.

85 Freiberger, Interview, 2009; Freiberger, Interview, 2017.

86 Baader, Interview, 2012; Baader, Telephone Interview, 2017; Institut Theresienstädter Initiative and Dokumentationsarchiv des österreichischen Widerstandes (DÖW), ed., *Theresienstädter Gedenkbuch: Österreichische Jüdinnen und Juden in Theresienstadt 1942*–*1945* (Prague: Institut Theresienstädter Initiative, 2005), 319. For more information on the Theresienstadt ghetto, see footnote 83.

87 Cornelia Essner, *Die “Nürnberger Gesetze” oder: Die Verwaltung des Rassenwahns 1933–1945* (Paderborn: Schöningh, 2002), 385–6, 419*–*20; Hilberg, *Vernichtung*, 436*–*45; Noakes, “Development of Nazi Policy,” 291*–*354; Gruner, *Rosenstraße*, 178–81; John A.S. Greenville, “Die ‘Endlösung’ und die ‘Judenmischlinge’ im Dritten Reich,” in *Das Unrechtsregime: Internationale Forschung über den Nationalsozialismus*, vol. 2, ed. Ursula Büttner (Hamburg: Christians, 1986), 91*–*121.

88 Dieter J. Hecht, Michaela Raggam-Blesch, and Heidemarie Uhl, eds., *Letzte Orte vor der Deportation: Die Wiener Sammellager 1941/42* (Vienna: Mandelbaum, forthcoming).

89 In the course of this paper, the term *Mischling* is only used for children of a Jewish and a non-Jewish parent *(Mischlinge of the first degree)*. See footnote 49.

90 Deportation guidelines from 20 February 1943 in: Gruner, *Rosenstraße*, 51*–*2, 135; Adler, *Der verwaltete Mensch*, 188, 199*–*200. These deportation guidelines were never published, since they were considered “top secret” *(“Geheime Reichssache”)*. Members of “mixed families” therefore had no precise knowledge of the key factors of their safeguarding.

91 Raggam-Blesch, “Alltag unter prekärem Schutz,” 297*–*8. See also i.a.: Christa Hämmerle and Li Gerhalter, eds., *Apokalyptische Jahre. Die Tagebücher der Therese Lindenberg 1938 bis 1946* (Vienna: Böhlau, 2010), 79–90.

92 Freiberger, Interview, 2009; Freiberger in: DÖW, *Erzählte Geschichte*, 197*–*205, 201.

93 Deportation guidelines usually named the destination of the transport, sometimes the number of people that were to be deported and then included a detailed definition of who was going to be deported and who would be exempt. Jewish members of “mixed marriages” and *Geltungsjuden* were only exempt under certain conditions that varied slightly over time. See for example Gruner, *Rosenstraße*, 51*–*2, 135; Gottwaldt and Schulle, *Judendeportationen*, 56–8, 140–4.

94 Alois Brunner – head of the Vienna *Zentralstelle für jüdische Auswanderung* and in charge of deportations – was sent to Berlin to speed up the deportation process there as well. Before the start of the *Fabrik Aktion*, however, he had already been sent on to Thessaloniki. Hans Safrian, *Die Eichmann-Männer* (Vienna: Europa Verlag, 1993), 189–92, 233–8; Hilberg, *Vernichtung*, 484–5; Gruner, *Rosenstraße*, 63.

95 Gruner, *Rosenstraße*, 95–117; Nathan Stolzfus, *Resistance of the Heart: Intermarriage and the Rossenstrasse Protest in Nazi Germany* (New York and London: W. W. Norton, 1996).

96 “Tagesbericht der Gestapo Wien,” USHMM, RG–17.035, TB 8 (26–28 October 1943), 3–4. DBSHOA: http://www.doew.at/erinnern/personendatenbanken/shoah-opfer (accessed 3 June 2019).

97 “Tagesbericht der Gestapo Wien,” USHMM, RG–17.035, TB 5 (12–15 February 1943), 5–6. DBSHOA: http://www.doew.at/erinnern/personendatenbanken/shoah-opfer (accessed 3 June 2019).

98 “Statistik der Staatspolizei(leit)stelle Wien für den Monat Januar 1944,” Attachment, “Tagesbericht der Gestapo Wien,” USHMM, RG–17.035, TB 1 (1–6 January 1944); Raggam-Blesch, “Survival of a Peculiar Remnant,” 212*–*3.

99 Name anonymized on request of the family.

100 VHA Interview (38836), 1997. Translation from the original German by the author.

101 *Mischlinge of the first degree;* see footnote 49.

102 Walk, *Sonderrecht*, 405.

103 Büttner, *Die Not der Juden teilen*, 65–6; Wolf Gruner, *Zwangsarbeit und Verfolgung: Österreichische Juden im NS-Staat 1938–45* (Vienna: Studienverlag, 2000), 282–3.

104 *Mischlinge of the first degree;* see footnote 49.

105 Baader, Interview, 2012.

106 There is differing information on the exact date of this edict. While Gruner dates the RSHA edict on 15 January, Gottwald and Schulle indicate the date with 19 January 1945. Walk, however, quotes a deportation ruling from a local Gestapo office in Rheinland–Pfalz (10 January 1945) – predating the RSHA edict, which he dates on 13 January 1945. Gruner, *Rosenstraße*, 187; Gottwaldt and Schulle, *Judendeportationen*, 366; Walk, *Sonderrecht*, 406.

107 While the official reason was the approaching front, the local Nazi officials were already aware of the imminent defeat and might have also considered strategies that would cast themselves in a more favourable light. Herbert Rosenkranz, *Verfolgung und Selbstbehauptung: Die Juden in Österreich 1938–1945* (Vienna: Herold, 1978), 309*–*10; Gruner, *Rosenstraße*, 187*–*8.

108 Only in 1997 were persons who had been endangered alone by virtue of belonging to a group, such as children of intermarried families classified as *Mischlinge* during the Nazi regime, recognized as victims in the meaning of the law. This recognition was the basis for so called gesture payments by the National Fund of the Republic of Austria for Victims of National Socialism: https://www.nationalfonds.org/victims-recognition.html (accessed 30 May 2019).

109 Baader, Interview, 2012; Baader, Telephone Interview, 2017; Philipp Peyman Engel, “Ruhestand? Nur formal,” *Jüdische Allgemeine*, 22 October 2009, https://www.juedische-allgemeine.de/allgemein/ruhestand-nur-formal/ (accessed 3 June 2019).

110 This was a stipulation of her ex-husband during the divorce proceedings in the 1960s, which were not devoid of antisemitic undertones. Freiberger, Interview, 2009; Freiberger, Interview, 2017.

111 VHA Interview (38836), 1997; Helga Feldner-Busztin, interview by the author on 14 September 2016 in Vienna.

112 Lotte Freiberger, *Erinnerungen*, DÖW 22103, undated manuscript, 9. Translation from the original German by the author.

